# Neuroprotective Effects of Platonin, a Therapeutic Immunomodulating Medicine, on Traumatic Brain Injury in Mice after Controlled Cortical Impact

**DOI:** 10.3390/ijms19041100

**Published:** 2018-04-06

**Authors:** Ting-Lin Yen, Chao-Chien Chang, Chi-Li Chung, Wen-Chin Ko, Chih-Hao Yang, Cheng-Ying Hsieh

**Affiliations:** 1 Department of Medical Research, Cathay General Hospital, Taipei 22174, Taiwan; d119096015@tmu.edu.tw; 2 Department of Pharmacology, College of Medicine, Taipei Medical University, Taipei 11031, Taiwan; change@seed.net.tw (C.-C.C.); chyang@tmu.edu.tw (C.-H.Y.); 3 Department of Cardiology, Cathay General Hospital, Taipei 10630, Taiwan; wckcgh@ms17.hinet.net; 4 Division of Pulmonary Medicine, Department of Internal Medicine, Taipei Medical University Hospital, Taipei 11031, Taiwan.; clchung@tmu.edu.tw; 5 School of Respiratory Therapy, Division of Thoracic medicine, Department of Internal Medicine, School of Medicine, College of Medicine, Taipei Medical University, Taipei 11031, Taiwan

**Keywords:** traumatic brain injury, platonin, neuroinflammation, microglial activation, free radical

## Abstract

Traumatic brain injury (TBI) is one of the leading causes of mortality worldwide and leads to persistent cognitive, sensory, motor dysfunction, and emotional disorders. TBI-caused primary injury results in structural damage to brain tissues. Following the primary injury, secondary injuries which are accompanied by neuroinflammation, microglial activation, and additional cell death subsequently occur. Platonin, a cyanine photosensitizing dye, has been used to treat trauma, ulcers, and some types of acute inflammation. In the present study, the neuroprotective effects of platonin against TBI were explored in a controlled cortical impact (CCI) injury model in mice. Treatment with platonin (200 µg/kg) significantly reduced the neurological severity score, general locomotor activity, and anxiety-related behavior, and improved the rotarod performance of CCI-injured mice. In addition, platonin reduced lesion volumes, the expression of cleaved caspase-3, and microglial activation in TBI-insulted brains. Platonin also suppressed messenger (m)RNA levels of caspase-3, caspase-1, cyclooxygenase-2, tumor necrosis factor-α, interleukin-6, and interleukin-1β. On the other hand, free radical production after TBI was obviously attenuated in platonin-treated mice. Treatment with platonin exhibited prominent neuroprotective properties against TBI in a CCI mouse model through its anti-inflammatory, anti-apoptotic, and anti-free radical capabilities. This evidence collectively indicates that platonin may be a potential therapeutic medicine for use with TBIs.

## 1. Introduction

Traumatic brain injury (TBI) is defined as damage to the brain resulting from an external mechanical force, such as that caused by rapid acceleration or deceleration, blast waves, crushing, impacts, or penetration by a projectile [1]. Over the past decade, TBI has been one of the main causes of mortality and one of the most serious public health problems worldwide. TBI not only causes a burden to medical resources but also contributes to approximately one-third of all injury-related deaths [2,3]. Persistent dysfunctions after TBI include cognitive, sensory, motor dysfunction, and emotional function disorders. Patients who suffer from TBI may confront excruciating pain for a few days to their entire lifetime. Consequently, these issues affect individuals and have lasting effects on their families and communities [2,4]. Brain damage caused by TBI is usually subdivided into primary and secondary injuries. The primary injury results in structural damage to the brain that occurs after exposure to an external force and includes contusions, subsequent damage to blood vessels (hemorrhaging, edema, and hypoxia), and diffuse axonal injury. Following the primary injury, secondary brain injuries occur through a complex cascade of biochemical and cellular changes that are accompanied by neuroinflammation, reactive oxygen species (ROS) generation, and additional cell death for a few minutes to many months [5,6].

Microglia are resident immune cells of the central nervous system and play a critical role in neuroinflammation. After TBI, microglia become highly activated and deliver cytokines and chemokines, which leads to apoptosis, neuroinflammation, and neurodegeneration due to neuron trauma [7,8]. Apoptosis of neurons and glia results in the pathology of TBI in both humans and animals [9]. The participation of caspase-3 in the neuronal apoptotic process after TBI has been previously examined [10,11]. Caspase-3 activation leads to cleavage of cytoskeletal proteins and proteolysis of DNA repair proteins, ultimately causing apoptosis and cell loss. Therefore, the abrogation of caspase-3 activation in brain tissues after TBI may help diminish the death of brain cells and maintain better neurological outcomes [12]. Previous studies have revealed that, in the postmortem human brain, messenger (m)RNA levels of proinflammatory cytokines, such as interleukin (IL)-6, IL-8, tumor necrosis (TNF)-α, and IL-1β, significantly increase in the brain following TBI insult [13]. Reductions in IL-1β, IL-6, monocyte chemoattractant protein (MCP)-1, and macrophage inflammatory protein (MIP)-2 in the injured brain revealed the neuroprotective effect in a controlled cortical impact (CCI) animal study [14]. In addition, experimental and clinical studies have provided additional evidence that neuroinflammation in TBIs involves the upregulation of ROS, cyclooxygenase (COX) enzyme, prostaglandin E2 (PGE_2_), and caspase-1, which contribute to cellular damage and subsequent apoptosis [15,16,17]. ROS scavengers and COX-2 inhibitors were suggested for use in treating neurological disorders such as stroke and TBIs [15,18], which indicated that ROS and COX-2 might be important targets of TBI therapy.

Platonin, a photosensitive trithiazolepentamethine cyanine dye, has been reported to have antioxidant, antimicrobial, and immunomodulatory activities [19,20,21] and is used for treating immune diseases [21]. Previous studies have indicated that platonin can moderate circulatory failure and downregulate mortality in septic rats [22] and attenuate sepsis-caused loss of an intact blood–brain barrier (BBB) [23]. Notably, the neuroprotective effect of platonin was demonstrated in a mouse model of ischemic stroke. Furthermore, the neuroprotective ability of platonin in ischemic brain injury may be attributed to its anti-inflammatory, antithrombotic, and free radical-scavenging effects [24].

On the basis of the anti-inflammatory and neuroprotective properties of platonin, which are intimately related to brain injuries, we hypothesized that platonin might be a potential therapeutic agent against TBIs. Moreover, in consideration of the critical role of the formation of excessive free radicals in the pathology of TBI, the in vivo scavenging activity of platonin against ROS was determined in the present study.

## 2. Results

### 2.1. Platonin Improves Neurobehavioral Functions in Mice Subjected to TBI

TBI has been known to lead to several long-term harmful symptoms including cognitive, sensory, motor dysfunction, and emotional disorders [2,4]. In order to determine the neuroprotective potential of platonin against TBI, a CCI animal model, a widely used animal model in TBI studies [25], was utilized to mimic pathological phenomena of TBI in this study.

**Figure 1:**
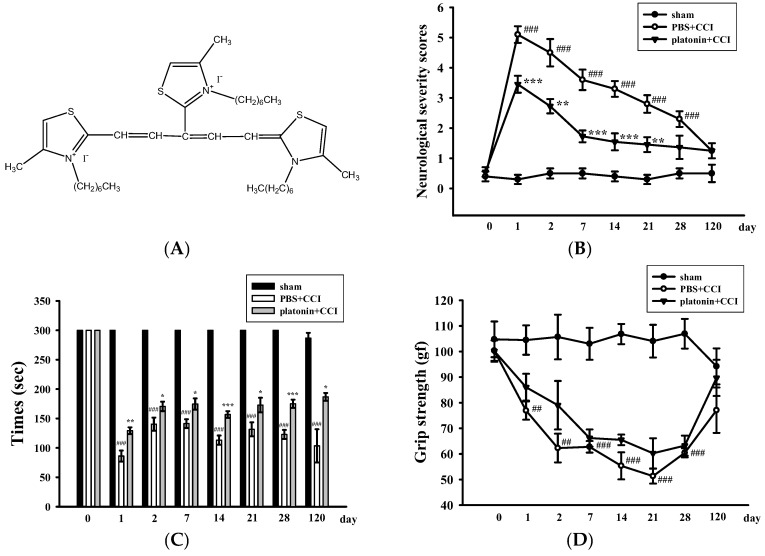

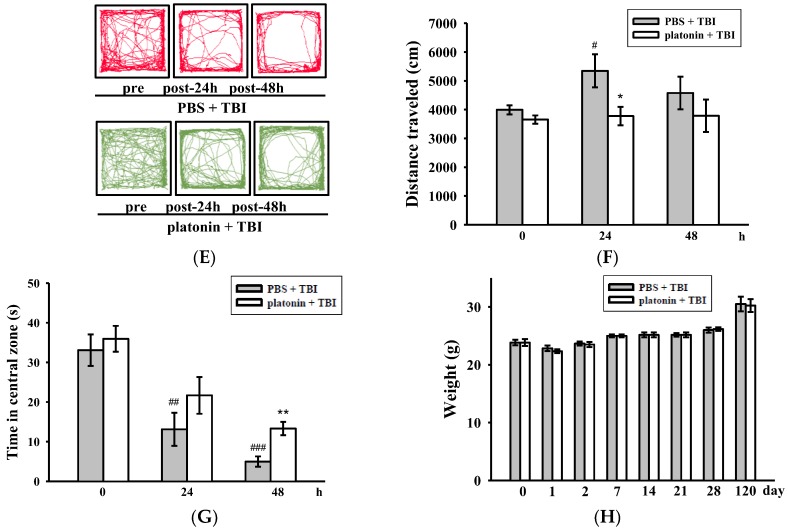
Alleviation of neurobehavioral deficits after platonin treatment in a mouse traumatic brain injury (TBI) model. Mice were treated with an isovolumetric solvent control (phosphate-buffered saline (PBS), intraperitoneally (ip)) or platonin (200 µg/kg, ip) for 30 min and then subjected to TBI). (**A**) The chemical structure of platonin. Assays of neurobehavioral functions including neurological severity scoring (**B**), a rotarod test (**C**), and a grip strength test (**D**) were performed before and 1, 2, 7, 14, 21, 28, and 120 days after TBI. All data are presented as the mean ± SEM (*n* = 6). ^##^ *p* < 0.01, ^###^ *p* < 0.001, compared to the sham group; * *p* < 0.05, ** *p* < 0.01 and *** *p* < 0.001, compared to the PBS group. Locomotor activity (**E**,**F**) and anxiety-like behavior (**G**) were evaluated before and 24 and 48 h after TBI. All data are presented as the mean ± SEM (*n* = 10). ^#^ *p* < 0.05, ^##^ *p* < 0.01 and ^###^ *p* < 0.001, compared to the presurgery group; * *p* < 0.05 and ** *p* < 0.01, compared to the PBS group. (**H**) Body weight of mice only treated with the isovolumetric solvent control (PBS, ip) or platonin (200 µg/kg, ip) after TBI, recorded for 0–120 days.

Neurological deficits were examined and scored on an 18-point scale before and 1, 2, 7, 14, 21, 28, and 120 days after the TBI. The varieties of neurological severity scoring of different groups are illustrated in Figure 1B. After the TBI, neurological severity scoring obviously increased compared to those before the TBI; nevertheless, treatment with platonin (200 µg/kg) significantly ameliorated these increased scores (^###^ *p* < 0.001; *n* = 6, compared to the sham group; ** *p* < 0.01 and *** *p* < 0.001; *n* = 6, compared to the phosphate-buffered saline (PBS) group). Rotarod tests and grip strength tests are widely used to evaluate sensorimotor functions in experimental models of TBI [26,27]. Figure 1C shows that the TBI resulted in a significant impairment in the rotarod test, and the decrease in the rotarod duration was reversed in the platonin-treated (200 µg/kg) group. (^###^ *p* < 0.001; *n* = 6, compared to the sham group; * *p* <0.05, ** *p* <0.01 and *** *p* < 0.001; *n* = 6, compared to the PBS group). In addition, mice that had received the CCI exhibited decreased grip strength compared to control mice (Figure 1D). Although it did not reach statistical significance, there was a trend showing that treatment with platonin (200 µg/kg) may ameliorate the impairment of grip force.

On the other hand, an open field test [28] was used to measure the general locomotor activity (LMA) and anxiety-like behavior in the present study. TBI significantly induced LMA in the vehicle-treated group at 24 h (^#^ *p* < 0.05; *n* = 10, compared to the pre-operated group); treatment with 200 µg/kg platonin extensively reversed the hyperactivity of LMA that occurred at 24 h after the TBI (* *p* < 0.05; *n* = 10, compared to the PBS group; Figure 1E,F). In addition, the time spent in the central zone of the open field decreased in mice with TBI. However, platonin-treated animals showed more extended times in the central zone than did vehicle-treated animals at 48 h after the TBI (^###^ *p* < 0.001; *n* = 10, compared to the 0 h group; ** *p* < 0.01; *n* = 10, compared to the PBS group; Figure 1G).

The body weight and mortality of mice used in this study were recorded during the 0–120 day period. Neither the vehicle (PBS) nor the platonin-treated (200 µg/kg, one-dose treatment on the first day) groups revealed a significant difference in body weight (Figure 1H) or mortality during the experimental period.

### 2.2. Platonin Attenuates Lesion Volumes and Caspase-3 Activation in Mice Subjected to TBI

**Figure 2:**
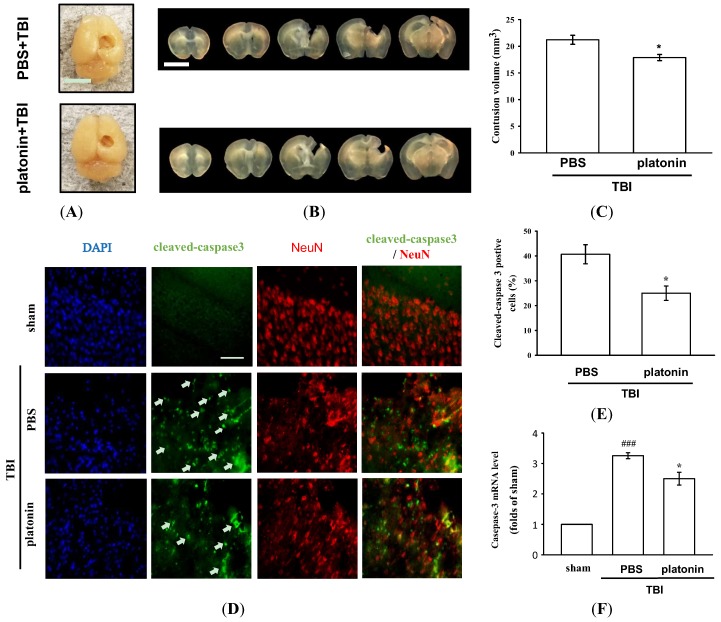
Mitigation of TBI-induced brain contusion volume and caspase-3 activation by platonin treatment in mice. (**A**) The photos represent the TBI impact location and exterior of brains of vehicle- (phosphate-buffered saline (PBS), ip) and platonin-treated (200 µg/kg, ip) mice on the 28th day after the TBI. The white bar indicates 5 mm. (**B**) Coronal brain sections (1 mm thick) showing the lesion volume, and quantitative analytical results are illustrated (**C**). The white bar indicates 5 mm. Data are presented as the mean ± SEM (*n* = 4). * *p* < 0.05, compared to the PBS group. (**D**,**E**) Mice were treated with the isovolumetric solvent control (PBS, ip) or platonin (200 µg/kg, ip) for 30 min and then subjected to TBI. The expression of cleaved-caspase-3 and NeuN was determined at 24 h after the impact by fluorescence microscopy as described in “Materials and Methods.” Fluorescence images are typical of those obtained in three separate experiments demonstrating caspase-3 activation (arrows) near the lesion portion in mice subjected to the TBI. Blue depicts the nucleus, green depicts cleaved caspase-3, and red depicts NeuN. The white bar indicates 75 µm. (**F**) The mRNA from contralateral brain homogenates at 24 h after TBI subjected to an RT-qPCR assay. Data are presented as the mean ± SEM (*n* = 6). ^###^ *p* < 0.001, compared to the sham group; * *p* < 0.05, compared to the PBS group.

The histological assessment of lesion volume of brain tissues was conducted 28 days after the TBI. No groups exhibited pathological changes in the contralateral brain site, whereas apparent potholes were revealed in the ipsilateral site (Figure 2A). As shown in Figure 2B, serial brain sections after the TBI showed severe contusion injuries, with substantial tissue loss in the cortex and damage to the hippocampal region. Treatment with platonin (200 µg/kg) resulted in a significant reduction (Figure 2A–C) in the cortical lesion volume; platonin-treated mice (17.88 ± 0.57 mm^3^) revealed an approximate 14% reduction in brain damage compared to vehicle-treated mice (21.21 ± 0.85 mm^3^). In the microscopic analysis (Figure 2D,E), cleaved caspase-3 expression had obviously increased at 24 h in mice exposed to the TBI, and treatment with platonin (200 µg/kg) potently attenuated the expression of cleaved caspase-3. Furthermore, the induction of caspase-3 mRNA expressions in TBI mice was significantly attenuated by treatment with platonin (Figure 2F) (^###^ *p* < 0.001; *n* = 6, compared to the sham group; * *p* < 0.05; *n* = 6, compared to the PBS group).

### 2.3. The Anti-Neuroinflammatory Effects of Platonin in Mice Subjected to TBI

**Figure 3:**
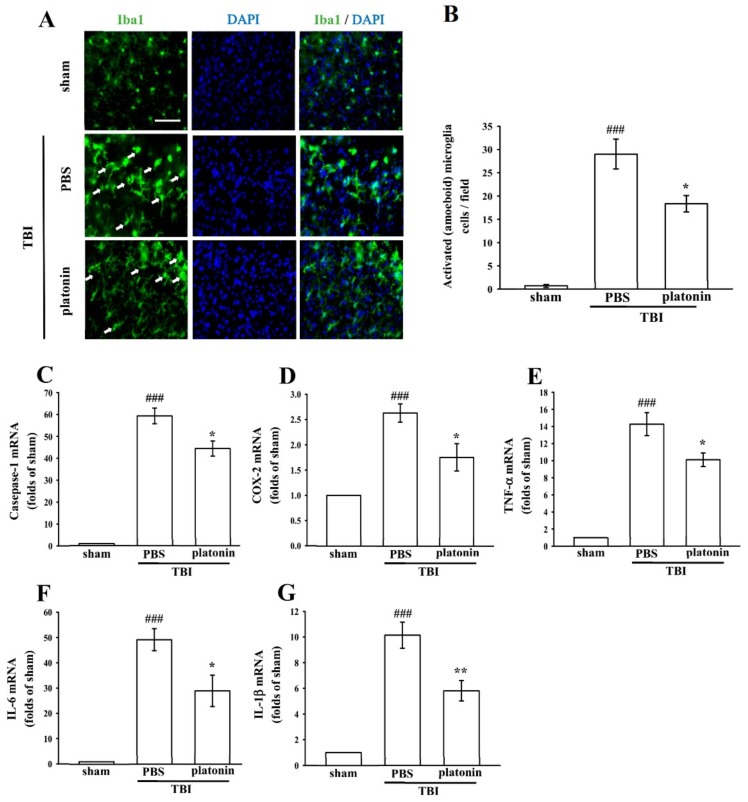
Treatment with platonin suppressed microglial activation and mRNA expressions of cyclooxygenase (COX)-2, caspase-1, and inflammatory cytokines in mice after a TBI. Mice were treated with the isovolumetric solvent control (phosphate-buffered saline (PBS), ip) or platonin (200 µg/kg, ip) for 30 min and then subjected to TBI. Brain slices were harvested at 24 h after surgery. (**A**) The expression of the ionized calcium-binding adaptor molecule (Iba1) was determined by a fluorescence microscopic analysis as described in “Materials and Methods.” Fluorescence images are typical of those obtained in three separate experiments demonstrating expression of the Iba1 near the lesion portion of mice subjected to TBI. Blue depicts the nucleus, and green depicts the Iba1. The white bar indicates 75 µm. (**B**) The number of activated microglia (arrows) was quantified, and data are illustrated. Data are presented as the mean ± SEM (*n* = 3). ^###^ *p* < 0.001, compared to the sham group; * *p* < 0.05, compared to the PBS group. mRNA from the contralateral brain was purified and subjected to an RT-qPCR assay to evaluate mRNA expressions of (**C**) caspase-1, (**D**) cyclooxygenase (COX)-2, (**E**) tumor necrosis factor (TNF)-α, (**F**) interleukin (IL)-6, and (**G**) IL-1β at 24 h post-TBI. Data are presented as the mean ± SEM (*n* = 5). ^###^ *p* < 0.001, compared to the sham group; * *p* < 0.05 and ** *p* < 0.01 compared to the PBS group.

Neuroinflammation is an important component of secondary injury after TBI and has been linked to various sequelae such as neurodegenerative diseases [29]. The hallmark of TBI-induced cerebral inflammatory responses includes microglial activation, blood–brain barrier (BBB) dysfunction, release of inflammatory cytokines, and recruitment of peripheral leukocytes into brain parenchyma [30,31]. To evaluate microglial activation in a mice model of TBI, we identified expression of the ionized calcium-binding adaptor molecule 1 (Iba1), a specific marker of microglia/macrophages, to distinguish morphological changes in microglia following the TBI. As shown in Figure 3A, resting (ramified) microglia were present in the sham-operated brain, while activated and amoeboid-form microglia had arisen in mouse brains at 24 h after being subjected to TBI. Treatment with platonin (200 µg/kg) significantly attenuated the expression of activated microglia (Figure 3B) (^###^ *p* < 0.05; *n* = 3, compared to the sham group; * *p* < 0.05; *n* = 3, compared to the PBS group).

We next evaluated expressions of several critical inflammatory factors, including COX-2, caspase-1, IL-1β, IL-6, and TNF-α in the present study. As shown in Figure 3C–G, mRNA levels of COX-2, caspase-1, IL-1β, IL-6, and TNF-α were significantly upregulated in brain tissues of mice subjected to TBI (*** *p* < 0.001; *n* = 5, compared to the sham control group). Increases in TBI-stimulated inflammatory factors were substantially inhibited by treatment with platonin (200 µg/kg) (^#^ *p* < 0.05, ^##^ *p* < 0.01; *n* = 5, compared to the PBS group) (Figure 3C–G).

### 2.4. Platonin Suppresses TBI-Induced Free Radical Formation in Mice through Upregulating Heme Oxygenase (HO)-1

**Figure 4:**
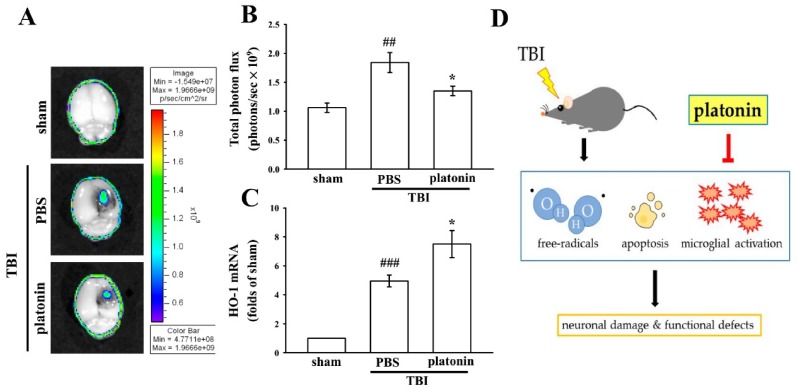
Reduction of free radicals and enhancement of heme oxygenase (HO)-1 expression after a TBI in mice treated with platonin. Mice were treated with the isovolumetric solvent control (phosphate-buffered saline (PBS), ip) or platonin (200 µg/kg, ip) for 30 min and then subjected to TBI. (**A**,**B**) The superoxide marker, dihydroethidium (DHE), was used to determine free radical production at 24 h post-TBI with the IVIS Imaging System. The color bar indicates the intensity of DHE fluorescence (total photon flux, photons/s). (**C**) mRNA from the contralateral brain was purified and subjected to an RT-qPCR assay to evaluate mRNA expression of HO-1 at 6 h post-TBI. Data are presented as the mean ± SEM (*n* = 5). ^##^ *p* < 0.01 and ^###^ *p* < 0.001, compared to the sham group; * *p* < 0.05 compared to the PBS group. (**D**) Diagram of the neuroprotective mechanism of platonin against TBI in mice.

Previous studies reported that TBI induced excessive free radical production, possibly exacerbating neuron injury by oxidizing lipids, proteins, and DNA, which contributes to further cellular damage and apoptosis [32]. In this study, the IVIS Imaging System was utilized to investigate the anti-free radical property of platonin in mice after TBI. Figure 4A reveals higher free radical production in brains of TBI-insulted mice than in those of the sham control group. The administration of platonin (200 µg/kg) markedly reduced TBI-induced free radical formation (Figure 4A). The statistical results are illustrated in Figure 4B (^##^ *p* < 0.01; *n* = 6, compared to the sham group; * *p* < 0.05, compared to the PBS group).

Heme oxygenase 1 (HO-1) is a phase II detoxifying/antioxidant enzyme and has been reported to have neuroprotective effects in brain injury. HO-1 provides protection in part through degrading its pro-oxidant substrate, heme, and releasing biliverdin and bilirubin as antioxidants [33]. Figure 4C demonstrates that HO-1 mRNA was upregulated in the vehicle-treated group at 6 h post-TBI (^#^ *p* < 0.05, compared to the sham group). Treatment with platonin (200 µg/kg) further enhanced HO-1 mRNA expression compared to that of the vehicle-treated group (* *p* < 0.05; *n* = 6, compared to the PBS group).

## 3. Discussion

Traumatic brain injury is a frequent and clinically highly heterogeneous neurological disorder with enormous economic consequences and burdens to both individuals and society. Neuroinflammation, apoptosis, and oxidative stress are considered major causes that link brain damage and neurodegeneration after TBI. Although advances in technology and treatment over the last few decades have improved the quality of life and longevity of patients with TBI, no effective drugs or preventative treatments currently exist. The present study used mouse CCI [34], a well-established experimental TBI model, to identify the neuroprotective effect of platonin. The results demonstrated that pretreatment of platonin exerts neuroprotective effects against TBI. Although posttreatment is the proper experimental design for clinical application in TBI or other acute brain injury such as ischemic stroke, this study still indicates the neuroprotective potential of platonin. In the future, we must change the timing of drug administration and perform the experiments in different animal species and animal models. In addition, even though the clinical application of platonin pretreatment is limited in acute TBI and ischemic stroke. The pretreatment of platonin may be advantageous in the prevention of chronic TBI or secondary ischemic stroke.

Psychiatric disturbances, including anxiety disorders and depressive disorders, are reported to occur significantly more frequently among individuals with TBI compared to healthy controls, regardless of the injury severity [35,36]. It was found that anxiety disorders were the most prevalent psychiatric outcome in patients at both 3 and 12 months post-injury [37]. In addition, TBI has been reported to induce hyperactive symptoms in both humans and mice [38,39]. Children with TBI may result in attentional deficits, response inhibition, and hyperactivity [40]. Due to the high incidence of TBIs and the common diagnosis of anxiety and hyperactivity following TBI, physical and pharmaceutical interventions should be identified to improve the psychosocial functioning and life quality of patients. We demonstrated that platonin significantly ameliorated general locomotor activity and anxiety-like behavior using an open field test. Figure 2E,F showed that the manifestation of hyperactivated locomotion after TBI had significantly decreased in platonin-treated mice. Anxiety-like behavior (Figure 2G) also improved in platonin-treated animals. These results suggest that platonin may have the effect of reducing anxiety disorders after TBI. However, based on the complexity of emotional diseases, more animal models should be used to confirm the impacts of platonin on anxiety. In this study, platonin was also found to inhibit expressions of proinflammatory cytokines after the TBI (Figure 3C–G). According to a previous study, neuroinflammation may be involved in the development and maintenance of anxiety-like behaviors after TBI [41]. The anti-neuroinflammatory effect may be an underlying mechanism in platonin’s improvement of TBI symptoms.

TBI-induced cell death is a major cause of neurological deficits and mortality. According to previous studies, cell apoptosis, microglial activation, and neuroinflammation are mediators of neuronal cell death and tissue loss after TBI [7,11,15,17]. After an acute TBI, apoptosis occurs in areas that are not severely affected by the injury; a biochemical cascade activates caspase-3 to destroy molecules that are required for cell survival and others that mediate a program of cell suicide [42]. Cell fragments and enzymatically cleaved chromosomes recruit activated microglia to the injured area for clearance of dying cells by phagocytosis [43]. Moreover, activated microglia secrete multiple neurotoxic factors including cytokines, chemokines, and ROS to drive progressive neuron damage [44]. In the present study, platonin showed an antiapoptotic property by reducing caspase-3 activation and caspase-3 mRNA expression after the TBI (Figure 2D–F). Microglial activation was also alleviated by treatment with platonin (Figure 3A). Moreover, mRNA expressions of IL-1β, IL-6, TNF-α, caspase-1, and COX-2 (Figure 3B–F) were also attenuated by platonin in mice subjected to TBI. As a result, treatment with platonin decreased the brain lesion volume (Figure 2A–C), and these results suggest that platonin may achieve neuroprotective effects by inhibiting neuron cell mortality and neuroinflammation after TBI.

In the past decade, major advances found that ROS are generated and harm the brain after TBI insult [16]. Platonin was shown to significantly attenuate increased ROS levels in collagen-induced platelets and the Fenton reaction system [24]. A previous study also indicated that endotoxemia was ameliorated by platonin through decreasing NO and free radical formation in a rat model [45]. In the present study, we further investigated the role of platonin in the endogenous antioxidant system. HO-1 is one of the phase II enzymes and was reported to have the most antioxidant response elements on its promoter, making it a highly effective therapeutic target for protecting against neurodegenerative diseases [46]. Figure 4A,B show that platonin downregulated free radical production at the injury lesion. In addition, a boost in HO-1 mRNA expression was observed in the platonin-treated group (Figure 4C). These findings are consistent with our previous study in which HO-1 played a vital role in neuroprotection against brain injury by inhibiting ROS production [33]. The neuroprotective effects of platonin after TBI might not only occur by scavenging ROS but also be due to moderation of the endogenous antioxidant system.

In conclusion, this study showed for the first time that platonin has therapeutic potential against brain injury after TBI. We evaluated the neuroprotective effects of platonin through a CCI animal model, and demonstrated that platonin treatment significantly improved neurobehavioral function and reduced the lesion volume after TBI. The antiapoptotic, anti-inflammatory, and free radical-inhibitory effects of platonin may contribute to its neuroprotective potential in TBI-insulted brains (Figure 4D). These results indicate that platonin may be a promising therapeutic drug for treating TBI.

## 4. Materials and Methods

### 4.1. Materials

Platonin was synthesized by and obtained from Gwo Chyang Pharmaceuticals (Tainan, Taiwan; Figure 1A). Primary antibody against cleaved-caspase-3 was purchased from Abcam (Cambridge, UK). The anti-Iba1 antibody was purchased from Wako Chemicals USA (Richmond, VA, USA). The anti-NeuN antibody was from Merck Millipore (Darmstadt, Germany). For immunostaining, CF488A donkey anti-mouse IgG and CF488A donkey anti-rabbit IgG were purchased from Biotium (Fremont, CA, USA). Dihydroethidium (DHE) was purchased from Cayman Chemical (Ann Arbor, MI, USA). Platonin was dissolved in phosphate-buffered saline (PBS) and stored at 4 °C.

### 4.2. Animals

Ten-week-old male C57BL/6 mice were purchased from BioLASCO (Taipei, Taiwan). All animal experiments and care procedures were approved by the Institutional Animal Care and Use Committee of Taipei Medical University. Before undergoing the experimental procedures, all animals were clinically normal and free from apparent infection, inflammation, or neurological deficits.

### 4.3. Controlled Cortical Impact (CCI) Injury

TBI was induced using a CCI device, and the injury procedure was performed as described previously [25]. Briefly, a mouse was anesthetized with isoflurane (induced at 4% and maintained at 3%) evaporated in a mixture containing 75% air and 25% oxygen, and then positioned in a stereotaxic frame with a heating pad to maintain its body temperature. After the mouse head was mounted, the scalp and fascia were retracted and a 3.5 mm circular craniotomy was performed on the right cerebral hemisphere (2.0 mm posterior to the bregma and 2.0 mm lateral to the sagittal suture) to expose the intact dura. For moderate-TBI induction, a 3-mm-flat impactor tip was used to impact the exposed dura (impact parameters: a velocity of 5.25 m/s, a depth of 2 mm, and a dwell time of 100 ms). After the CCI injury was performed, the scalp incision was sutured, and the mouse was placed on a heating pad until it had recovered from anesthesia. Mice were divided into three groups: (1) a sham-operated group; (2) a group treated with an isovolumetric solvent (PBS; intraperitoneally (ip)), followed by CCI; and (3) a group treated with platonin (200 µg/kg ip), followed by CCI. All treatments were administered 30 min before the CCI in all the groups except the sham-operated group, which received no injury.

### 4.4. Neurological Severity Examination

A neurological examination was performed on each mouse immediately before and at 1, 2, 7, 14, 21, and 28 days after the TBI. Neurological severity scores (NSSs) were derived using an 18-point sliding scale (normal score, 0; maximal deficit score, 18) [24]. The NSS examination includes motor tests (flexion of forelimb and hindlimb, head movements, and walking ability), reflex tests (pinna, corneal, and startle reflexes, and epileptic seizures), sensory tests (placing and proprioceptive tests), and balance tests (beam balance tests). In an NSS test, 1 point represents an inability to perform the test or the lack of a tested reflex (Table 1). Therefore, a higher score indicates a more severe injury.

### 4.5. Spontaneous Locomotor Activity and Rotarod Assessments

Before neurobehavioral testing, the mice were trained daily for 3 days. They were then trained to stay on an accelerating rotarod (4~40 rpm over 5 min, with increasing increments of 4 rpm at 30 s intervals) for three trials daily before being subjected to TBI surgery. Before and 24 and 48 h after the TBI, each mouse was placed in the center of the open field apparatus (50 × 50 × 40 cm). Locomotor activity (LMA) was automatically recorded with an activity monitor (Noldus Information Technology, Wageningen, the Netherlands) in the test period to count the total distance traveled, time spent in the center (25 × 25 cm), and the number of beam-break counts. After the LMA assessment, the rotarod performance was assessed to test the balance and coordination (UGO Basile, Varese, Italy). The rotarod was rotated from 4 to 40 rpm within 3 min. The time (in seconds) at which each mouse fell from the drum was determined for up to 300 s with a stopwatch and recorded.

### 4.6. Grip Strength Assessment

Neuromuscular strength was tested with grip strength tests. A grip strength meter (UGO Basile, Varese, Italy) was used to assess the forelimb grip strength. A mouse was lifted and held by its tail so that its forepaws could grasp a wire grid. The mouse was then gently pulled backward by its tail with its body parallel to the surface of the table until it released the grid. The peak force applied by the forelimbs of the mouse was recorded in gram-force (gf). Each mouse was tested three times, and the mean of the measured value was used for the statistical analysis.

### 4.7. Measurement of Brain Lesion Volume 

At 28 days after TBI, the mice were anesthetized and perfused with 4% paraformaldehyde in phosphate buffer (PB), and the brains were then transferred into 4% paraformaldehyde overnight and immersed by 30% sucrose for 24 h. Each brain was sliced into five 1-mm-thick slices and analyzed by utilizing the ImageJ 1.50i software (National Institutes of Health, Bethesda, MD, USA). The volume measurement was calculated by summation of the lesion areas: [(area of the intact contralateral left hemisphere) − (area of the intact ipsilateral right hemisphere)] × slice thickness (1 mm).

### 4.8. Immunofluorescent Staining of Brain Tissues

Animals of each group were euthanized and perfused with 4% paraformaldehyde in PB. Brains were post-fixed with 4% paraformaldehyde in PB overnight. Before sectioning, the brains were immersed in 30% sucrose in PB for cryoprotection. The brains were sliced in the coronal plane at 50 µm and permeabilized with PB containing 0.2% Triton X-100 and 6% donkey serum for 60 min. These slices were incubated overnight at 4 °C with primary antibodies including anti-caspase-3, anti-NeuN, and anti-Iba-1 antibodies. Subsequently, samples were washed three times with 0.2% Tween 20 in PB and then exposed to secondary antibodies overnight. The prepared slices were then counterstained with DAPI (30 mM) and mounted with mounting buffer (Vector Laboratories, Burlingame, CA, USA) on a glass coverslip. Samples were detected under an Evos FL Auto 2 imaging system using the LED fluorescence light cubes (Thermo Fisher Scientific, Waltham, MA, USA).

### 4.9. Real-Time Reverse-Transcription Quantitative Polymerase Chain Reaction (RT-qPCR)

Approximately 10 mg of fresh brain tissue was immediately collected from the ipsilateral (injured) hemisphere of a euthanized mouse and processed for a real-time RT-qPCR. Total RNA was isolated using the NucleoSpin RNA isolation kit (Macherey-Nagel, Düren, Germany). The purity and quality of RNA were confirmed by defining the ratio of absorbance levels at wavelengths of 260 and 280 nm (NanoDrop ND-1000, Thermo Fisher Scientific). A sample of 1 µg of total RNA was treated with the high-capacity cDNA reverse transcription kit (Thermo Fisher Scientific) and reverse-transcribed into complementary (c)DNA. For mRNA measurements, diluted cDNA was amplified using a QuantiNova SYBR Green PCR Kit (Qiagen, Hilden, Germany) in a Rotor-Gene Q 2plex HRM Platform (Qiagen). Reaction conditions were carried out for 35~40 cycles (5 min at 95 °C, 5 s at 95 °C, and 10 s at 60 °C). Quantified values of RNA were normalized with those of glyceraldehyde 3- phosphate dehydrogenase (GAPDH). The primers used for the RT-qPCR assay were as follows: caspase-3: 5′-GGGCCTGTTGAACTGAAAAA-3′ (forward) and 5′-CCGTCCTTTGAATTTCTCCA-3′ (reverse); caspase-1: 5′-AGGAATTCTGGAGCTTCAATCAG-3′ (forward) and 5′-TGGAAATGTGCCATCTTCTTT-3′ (reverse); COX-2: 5′-CCACTTCAAGGGAGTCTGGA-3′ (forward) and 5′-AGTCATCTGCTACGGGAGGA-3′ (reverse); TNF-α: 5′-TCTTCTGTCTACTGAACTTCGG-3′ (forward) and 5′-AAGATGATCTGAGTGTGAGGG-3′ (reverse); IL-6: 5′-CCTCTCTGCAAGAGACTTCCATCCA-3′ (forward) and 5′-GGCCGTGGTTGTCACCAGCA-3′ (reverse); IL-1β: 5′-AACCTGCTGGTGTGTGACGTTC-3′ (forward) and 5′-CAGCACGAGGCTTTTTTGTTGT-3′ (reverse); HO-1: 5′-GCACTATGTAAAGCGTCTCC-3′ (forward) and 5′-GACTCTGGTCTTTGTGTTCC-3′ (reverse); GAPDH: 5′-AGACAGCCGCATCTTCTTGT-3′ (forward) and 5′-CTTGCCGTGGGTAGAGTCAT-3′ (reverse).

### 4.10. Evaluation of Free Radical Production in Brain Tissues

Dihydroethidium (DHE) was used to detect brain damage-induced free radical production [33]. Sham-operated and TBI-insulted (including the PBS and platonin-treated groups) mice were administered DHE (0.5 mg in 100 µL of DMSO) through a tail vein injection 23.5 h after TBI treatment. The mice were euthanized 24 h after the TBI and perfused with cold saline from the left ventricle for 10 min. Subsequently, whole-brain tissues were immediately viewed under the IVIS Imaging System 200 Series (Xenogen Corporation, Alameda, CA, USA) to monitor ROS production. They were from separate groups of animals, and data are expressed as the total photon flux in a region of interest and expressed as photons per second.

### 4.11. Statistical Analysis

Data are expressed as the mean ± standard error of the mean (SEM) of the results and are accompanied by the number of observations. The normality of the data was first tested using the Kolmogorov–Smirnov test. Continuous variables were compared using an analysis of variance (ANOVA). When the analysis indicated significant differences among group means, each group was compared using the Newman–Keuls method. *p* < 0.05 was considered statistically significant.

## Figures and Tables

**Table 1:** Neurological severity scores (NSSs).

Tests	Points
**Motor tests**	6
Raising mice by the tail (normal = 0; maximum = 3)	3
Flexion of forelimb	1
Flexion of hindlimb	1
Head moved >10° to vertical axis within 30 s	1
Placing mice on the floor (normal = 0; maximum = 3)	3
Normal walk	0
Inability to walk straight	1
Circling toward the paretic side	2
Fall down to the paretic side	3
**Sensory tests**	2
Placing test (visual and tactile test)	1
Proprioceptive test (deep sensation, pushing the paw against the table edge to stimulate limb muscles)	2
**Beam balance tests (normal = 0; maximum = 6)**	6
Balances with steady posture	0
Grasps side of beam	1
Hugs the beam and one limb falls down from the beam	2
Hugs the beam and two limbs fall down from the beam, or spins on beam (>60 s)	3
Attempts to balance on the beam but falls off (>40 s)	4
Attempts to balance on the beam but falls off (>20 s)	5
Falls off: No attempt to balance or hang on to the beam (<20 s)	6
**Reflexes absent and abnormal movements**	4
Pinna reflex (head shake when touching the auditory meatus)	1
Corneal reflex (blink when lightly touching the cornea with cotton)	1
Startle reflex (motor response to a brief noise from snapping a clipboard paper	1
Seizures, myoclonus, myodystony	1
**Maximum points**	18
